# Influence of two different resection techniques (conventional liver resection versus anterior approach) of liver metastases from colorectal cancer on hematogenous tumor cell dissemination – prospective randomized multicenter trial

**DOI:** 10.1186/1471-2482-8-6

**Published:** 2008-03-05

**Authors:** T Schmidt, M Koch, D Antolovic, C Reissfelder, FH Schmitz-Winnenthal, NN Rahbari, J Schmidt, CM Seiler, MW Büchler, J Weitz

**Affiliations:** 1 Department of Surgery, University of Heidelberg, Heidelberg, Germany

## Abstract

**Background:**

Surgical hepatic resection remains the treatment of choice for patients with liver metastases from colorectal cancer despite the use of alternative therapeutic strategies. Although this procedure provides long-term survival in a significant number of patients, 50–75% of the patients develop intra- and/or extrahepatic recurrence. One possible reason for tumor recurrence may be intraoperative hematogenous tumor cell dissemination due to mechanical manipulation of the tumor during hepatic resection. Surgical technique may have an influence on hematogenous tumor cell spread. We hypothesize that hematogenous tumor cell dissemination may be reduced by using the anterior approach technique compared to conventional liver resection.

**Methods/Design:**

This is a multi-centre prospective randomized controlled, superiority trial to compare two liver resection techniques of liver metastases from colorectal cancer. 150 patients will be included and randomized intraoperatively after surgical exploration just prior to resection. The primary objective is to compare the anterior approach with the conventional liver resection technique with regard to intraoperative haematogenous tumor cell dissemination. As secondary objectives we examine five year survival rates (OS and DFS), blood loss, duration of operation, requirement of blood transfusions, morbidity rate, prognostic relevance of tumor cell detection in blood and bone marrow and the comparison of tumor cell detection by different detection methods.

**Conclusion:**

This trial will answer the question whether there is an advantage for the anterior approach technique compared to the conventional resection group with regard to tumor cell dissemination. It will also add further information about prognostic differences, safety, advantages and disadvantages of each technique.

**Trial registration:**

Current controlled trials – **ISRCTN45066244**

## Background

Despite the use of alternative therapy strategies surgical resection of colorectal liver metastases remains the treatment of choice and is associated with long-term survival in a significant number of patients [1,2]. After curative resection of colorectal liver metastases the 5-year survival rate ranges from 20% to 51% depending on preoperative selection criteria and published series [3]. However, up to 75% of resected patients will develop extra- or intrahepatic tumor recurrence [4]. One possible cause for this tumor recurrence may be intraoperative hematogenous tumor cell dissemination due to mechanical manipulation of the tumor during hepatic resection [5].

Recently, RT-PCR based protocols were developed for the detection of disseminated tumor cells [6]. We developed a sensitive and specific CK 20 RT-PCR system for detection of disseminated colorectal cancer cells in blood, bone marrow and lymph nodes [7-9]. By using the CK 20 RT-PCR, we demonstrated that there is a significantly enhanced intraoperative hematogenous tumor cell spread during resection of primary tumor and liver metastases from colorectal cancer [10,11]. In 12 of 38 patients (31,6%) with colorectal liver metastases tumor cells were detected only during or during and after surgery, possibly indicating an intraoperative tumor cell dissemination [9]. Statistical analysis revealed an increased risk for intraoperative tumor cell dissemination during major liver resection for metastases compared with minor liver resections or resection of the primary tumor (Odds ratio = 3.07; p = 0.05). These observations are in accordance with the concept of intraoperative tumor cell dissemination, because a higher degree of intraoperative manipulation of the liver and tumor in more extensive resections should result in a higher incidence of tumor cell shedding. Recent follow up of the patients with resection of colorectal liver metastases has shown, that intraoperative detection of circulating tumor cells is associated with a worse prognosis [10].

Surgical technique might influence hematogenous tumor cell spread and patient's prognosis. Initially, Liu et al. showed in a retrospective study of patients with hepatocellular carcinoma a significantly improved survival rate for patients undergoing the anterior approach technique (with primary dissection of the parenchyma and without mobilization of the liver) compared to conventional liver resection [12]. This study was followed by a prospective randomized controlled study in which the use of the anterior approach technique for liver resection in large hepatocellular carcinomas was confirmed to have a significantly better overall survival compared to the use of the conventional technique[13].

The hypothesis of this study is that intraoperative hematogenous tumor cell dissemination could be reduced or prevented by using the anterior approach technique in resection of metastasis of colorectal cancer in the liver.

## Methods/design

### Aim of the study

The objective of this trial is to compare two different surgical techniques for the resection of colorectal liver metastases with regard to intraoperative hematogenous tumor cell dissemination, survival (overall and disease-free survival), blood loss, duration of resection, transfusion requirements, complications, relevance of tumor cell detection in the bone marrow and comparison of different tumor cell detection methods.

### Trial population

The anterior approach trial includes patients with the clinical diagnosis of metastatic colorectal cancer to the liver who are being planned for elective and potentially curative (R0) resection of the right liver lobe. This study includes patients over 18 years of age who are planned for right hepatectomy (removal of segments 5–8), extended right hepatectomy (removal of segments 5–8 and parts of segment 4) or right trisegmentectomy (removal of segment 4–8). Based on the preoperative imaging the surgeon has to determine that the liver metastases can be removed with both resection techniques. Patients with extrahepatic disease, liver cirrhosis and tumor positive lymph nodes in the hepatoduodenal ligament will be excluded from the trial. A detailed overview of all eligibility criteria are given in Table 1.

**Table 1:** Eligibility Criteria

**Inclusion criteria** (At study enrolment)
• Patients being considered for a potentially curative (R0) right hepatectomy (removal of segments 5,6,7,8), extended right hepatectomy (removal of segments 5,6,7,8, part of segment 4) or right trisegmentectomy (removal of segments 4,5,6,7,8) for colorectal liver metastases
• Age equal or greater than 18 years
• Absence of any psychological, familial, sociological or geographical condition potentially hampering compliance with the study protocol, follow-up schedules or from signing informed consent
• Written informed consent from each patient or the patient's legal guardian prior to entering the study
• No evidence of active or former concurrent malignant diseases (except non-melanous skin cancer)

**Exclusion criteria**

• Any extrahepatic disease, even if this will be resected concomitantly
• Liver cirrhosis
• Grossly positive lymph nodes in the hepatoduodenal ligament
• Positive margins after liver resection (R1) (Patients will be excluded from further follow up, the patient's data however will be recorded).
• Patients with an intraoperative blood loss of >= 2000 cc will be excluded from the analysis of tumor cell detection in blood samples but will be included in the rest of the analyses.

### Study design

The anterior approach trial is a registered [ISRCTN45066244], prospective intraoperatively randomized (expertise-based) multicenter trial of patients who will undergo elective resection for colorectal liver metastases. It is designed as a two-group parallel superiority study. As the primary objective of this study is the extent of intraoperative tumor cell dissemination we hypothesize that intraoperative tumor cell shedding can be reduced significantly in the anterior approach compared to the conventional approach group.

### Surgical Interventions

All surgical interventions in this trial follow standardised described techniques which will be shortly described below. A detailed description of all surgical techniques is also in the study protocol.

#### Incision lines

Abdominal incision can be achieved according to the preference of the surgeon. A complete exploration of the abdomen, including potential frozen sections (of suspicious lesions or lymph nodes) is performed to decide whether a curative resection is feasible. An intraoperative ultrasound evaluation of the left liver is performed. If both approaches seem possible for the surgeon randomization will be performed at this point.

#### Conventional approach

The liver will be mobilized from the retroperitoneum including the division of short venous branches to the inferior vena cava. In the next step the inflow control (either intra- or extrahepatic) is performed by ligation and division of right portal vein and hepatic artery. The outflow is controlled by ligation and division of the right hepatic vein, and possibly, middle hepatic vein. Parenchymal transection is performed according to surgeon's preference and local standards (e.g. stapler, Cusa, etc.). Optionally, Pringle manoeuvre can be performed.

#### Anterior approach

Extrahepatic inflow control is maintained by ligation and division of right portal vein and hepatic artery before any mobilization of the right liver lobe. A hanging liver manoeuvre is performed without manipulation of the right lobe [14]. Afterwards, parenchymal transaction is performed (with optional Pringle manoeuvre). This is followed by the outflow control with ligation and division of the right, and possibly, the middle hepatic vein. Finally, the short venous branches of the inferior vena cava will be divided and the liver will be mobilized out of the retroperitoneum.

Any deviation or change of the standardized operation techniques will be regarded as a protocol violation. For safety reasons, the operating surgeon is allowed to change the surgical technique at any time point during the operation.

### Sample acquisition

Blood sample: From each patient two central venous blood samples will be obtained. One blood sample (10 cc) will be drawn after induction of general anesthesia through a central venous catheter, which is routinely placed just before surgery. Immediately after resection of the liver metastases, 10 cc of central venous blood will again be taken. The intraoperative blood sample will be obtained without occlusion of the hepatoduodenal ligament. If a wedge or segmental liver resection or other organ resection is performed in addition to a right hepatectomy the intraoperative blood sample will be obtained after the right hepatectomy. Bone marrow sample: A bilateral bone marrow sample (each 10 cc) will be taken after induction of general anesthesia just prior to skin incision after disinfection from both iliac crests. Bone marrow sampling is optional for the patient.

Note: EDTA and not Heparin will be used as anticoagulant for bone marrow and blood samples.

### Sample size and statistical consideration

150 patients will be accrued to this study. Patients who underwent R1 resection and patients with an intraoperative blood loss of greater than 2000 cc will be excluded.

The sample size of this trial was calculated with regard to the previous results and data from our study on hematogenous tumor cell dissemination during resection of liver metastasis from colorectal cancer [10] and the study on the use of the anterior approach as a new liver resection technique in patients with hepatocellular carcinoma [12]. For statistical sample size calculation a 1:1 randomization in both treatment groups was assumed.

From the analysis of the previous data we expect to detect a difference of at least 30% in the proportion of intraoperative tumor cell dissemination between the two groups. The incidence of hematogenous tumor cell dissemination in the conventional group is reported to be 50–55% and it will be expected to be 15–20% in the anterior resection groups. To achieve a power of 90% (beta = 0.1) approximately 112 patients have to be recruited (two-sided test, Type I error of 5%). Due to the expectation that about 25% of all the patients will have circulating tumor cells at baseline we have to increase the total number of randomized patients to 150 patients (75 in each arm). With this sample size the study will have adequate power to address the primary objective.

### Randomisation, stratification and blinding

The study is conducted in a prospective randomized manner. After giving informed consent and inclusion into the study, patients will be randomized in the operating room after surgical exploration just prior to resection by using sealed opaque serial numbered envelopes. These envelopes will be supplied by the Clinical Trials Centre (KSC) of the Department of Surgery, University of Heidelberg. The patients will be randomized 1:1 into each arm of the two different liver resection techniques. As stratification factors, the participating institution and the Fong score will be used [15]. The Fong score predicts the risk of recurrence after surgical resection of colorectal metastases to the liver. Two strata will be used (Fong score 0–2 and 3–5).

### Endpoints

As primary endpoint, we choose the tumor cell detection rate in blood samples obtained intraoperatively using a CK 20 RT-PCR. The method is already established and this was the endpoint for the sample size calculation and further decision making. As secondary endpoints, overall survival and disease-free survival will be recorded during the follow-up in both groups. Further secondary endpoints are blood loss, duration time of resection, number of blood products transfused, prognostic relevance of tumor cell dissemination to the bone marrow, and postoperative complications. All these parameters will be collected prospectively as part of the study protocol.

### Laboratory workup

The processing of blood and bone marrow samples will be done in each participating institution using a standard protocol. After processing, samples will be stored at -80°C for further laboratory workup which will be centrally performed at the Department of Surgery, University of Heidelberg. The samples from participating institutions will be sent to the laboratory at the Department of Surgery, University of Heidelberg by overnight service on dry ice. The samples will be accompanied by positive controls to ensure quality of processing and mailing of the samples. Blood and bone marrow samples will be analyzed by a CK 20 RT-PCR as previously described [7].

#### RT-PCR

All PCR reactions will be accompanied by positive and negative controls to ensure adequate performance of the procedure. Two investigators independently judge the results of the PCR. In case of different judgment (which in case of RT-PCR virtually never happens), the PCR result will be confirmed by hybridization.

The laboratory workup of all samples will be centrally performed in the Department of Surgery, University of Heidelberg. The technician working up the sample and the physician analyzing the test results are blinded for clinical data of each patient

### Clinical evaluation

The clinical evaluation of the patients includes a preoperative, intraoperative and postoperative evaluation. In the preoperative evaluation the operating surgeon has to evaluate whether the liver metastasis or metastases can be resected by either technique. During the operation the operating surgeon can change the technique at any time during the procedure if necessary for the patients safety. In this case the patient will be removed from the study.

### Safety aspects and adverse advents

Both surgical techniques are well established procedures and no specific side effects are expected in addition to the known complications of liver resections. Furthermore, the responsible surgeon can change the procedure at any given time point during the operation.

All precautions will be used to minimize the potential risks of bone marrow puncture which are bleeding, hematoma and infection. Since the central venous catheter for blood sampling is routinely placed in the operating theatre, no potential adverse effects of the blood sampling are expected.

Adverse events will be recorded during the hospital stay and the follow up.

### Ethics and informed consent

The final protocol was approved by the ethics committee of the University of Heidelberg. Written informed consent is obtained from each patient in oral and written form before inclusion in the study. The investigator will not undertake any measures specifically required only for the clinical trial until valid consent has been obtained. This must be done in accordance with the national and local regulatory requirements.

Patients are informed about the strict confidentiality of their personal data within this trial, but their medical records may be reviewed for trial purposes by authorized individuals other than their treating physician.

Patients will be informed about the additional specific risk of a bone marrow aspiration, which is voluntarily and will not lead to exclusion from the study in case of refusal.

It will be emphasized that the participation is voluntary and that the patient is allowed to refuse further participation in the protocol whenever he/she wants. This will not prejudice the patient's subsequent care.

The informed consent procedure is in accordance with the ICH guidelines on Good Clinical Practice.

### Follow up

The patients will undergo routine follow-up (physical examination of the patients, tumor markers (CEA), abdominal ultrasound and/or CT/MRI, chest X-ray, colonoscopy) at least once a year. Disease free survival and overall survival will be recorded; at each time point a follow-up evaluation form will be completed.

As the prognostic impact of disseminated tumor cells is still unclear, the clinical management and postoperative treatment of the patients will not be influenced by the results of this study. Decisions about further adjuvant treatment (e.g. chemotherapy) of the patients are left to the discretion of the treating oncologist.

### Data management and quality assurance

This trial is coordinated by the Department of Surgery at the University of Heidelberg. The data regarding tumor cell detection in blood and bone marrow of all patients will be entered in a password-protected database at the Department of Surgery, University of Heidelberg. The clinical data of all included patients will be centrally collected in a database at the Department of Surgery, University of Heidelberg. To transfer the data to Heidelberg, the evaluation forms will be sent from the participating centers via overnight service. The clinical and laboratory data will be merged in the password-protected database at the Department of Surgery, University of Heidelberg, Germany.

All samples and clinical data will be tracked by means of a unique research tracking number that will not be related to any patient identifying information. The link between research tracking numbers and patient identifiers will be kept in a limited access database on a computer with password protection. Routine data quality reports will be generated to assess missing data and inconsistencies. Accrual rates and accuracy of evaluations and follow-up will be monitored periodically throughout the study period and potential problems will be brought to the attention of the study team for discussion and action. Random-sample data quality and protocol compliance audits will be conducted by the study team.

Only surgeons who performed 25 or more successful liver resections are allowed to recruit patients for this study. The records of the operations will be reviewed centrally in order to identify protocol violations.

### Analysis

We evaluate the influence of two different liver resection techniques on the incidence of intraoperative hematogenous tumor cell dissemination in patients with colorectal liver metastases. Patients with circulating tumor cells present in the bloodstream at baseline (before operation) will be excluded from the primary analysis, hence the primary endpoint is the presence of disseminated tumor cells after liver resection. Details of the timing of the different blood samples drawn from each patient are provided.

The analyses will be performed as intention to treat analyses.

An interim analysis regarding the incidence of intraoperative tumor cell dissemination between the two arms in this study will be undertaken after the randomization of the first 75 patients. If the p-value is < 0.005, the study will be terminated and it will be concluded that the anterior approach technique significantly reduces intraoperative tumor cell dissemination. If the p-value is > 0.65 the trial will be terminated and it will be concluded that there is no significant difference between the two techniques with regard to intraoperative tumor cell dissemination. For a p-value between 0.005 and 0.65, the study will be continued to the total number of 150 patients. The interim analysis is planned using the O'Brien-Fleming boundary with a provision to stop early in favor of the null hypothesis.

Primary comparison of the proportion of patients with disseminated tumor cells in the blood after liver resection will be carried out by Fisher's exact test.

Secondary endpoints as blood loss and duration time of resection will be evaluated by t-test, and amount of used blood units and complication rates will be evaluated by Fisher's exact test. Survival probabilities will be estimated by Kaplan-Meier method and groups will be compared using the log-rank test. A comparison of survival curves will be only feasible after long term follow-up, currently estimated as at least three years. Prognostic relevance of tumor cell detection in blood and bone marrow will be assessed using proportional hazards regression.

The Cochran's Q test will be performed to correlate PCR results with timing of blood collection.

Tumor cell detection rates in blood versus bone marrow samples will be compared with the Mc Nemar's test.

### Current status

At the current time point we have randomized 61 patients in 2 different centers. The duration of the trial is expected to be up to 3 more years. About 2–4 patients can be enrolled per month. The interim analysis will be performed during next year. Evaluation and reporting of the clinical and laboratory results will be done within 3 month of the end of recruitment and closing of the database.

## Discussion

The primary objective of this study is to compare two surgical resection techniques with regard to the extent of intraoperative hematogenous tumor cell dissemination. Tumor cell shedding into circulation during surgical manipulation is a long debated issue. Previous studies from our group demonstrated significantly enhanced hematogenous tumor cell dissemination during resection of primary tumor and liver metastases in patients with colorectal cancer [7-9]. Furthermore, we could show a prognostic impact of hematogenous tumor cell dissemination in patients with primary and metastatic colorectal cancer [16]. Retrospective and prospective data from Liu et al. indicated that the anterior approach technique provides a better survival compared to conventional resection for patients with hepatocellular carcinoma [11,12]. It remains unclear which aspect is mainly responsible for the superior oncological outcome of patients with hepatocellular carcinoma undergoing the anterior approach technique.

Therefore, we hypothesize that hematogenous tumor cell dissemination may be reduced by using the anterior approach technique compared to conventional liver resection. If this is true, tumor cell detection in blood and bone marrow may serve as a surrogate marker for assessment of oncological surgical procedures by detection of intraoperative hematogenous tumor cell dissemination. Furthermore, detection of hematogenous tumor cell dissemination could help to define a subgroup of patients who benefit from additional therapy (e.g. antibody or cytotoxic therapy) after extended surgical resection.

This hypothesis along with our above mentioned data regarding tumor cell dissemination were the basis for the conduction of this trial. This study is therefore a good example for translation of molecular data into a clinical trial.

## Competing interests

The author(s) declare that they have no competing interests.

## Authors' contributions

JW, MK, JS, MWB, CMS designed the study and contributed to manuscript preparation. JW, JS, MK, DA, CR, HSW, TS conduct the study. TS and NNR drafted the manuscript. All authors read and approved the final manuscript.

## Pre-publication history

The pre-publication history for this paper can be accessed here:


